# Numerical Studies of the Viscosity of Reacting Polyurethane Foam with Experimental Validation

**DOI:** 10.3390/polym12010105

**Published:** 2020-01-04

**Authors:** Kay Schäfer, Daisy Nestler, Jürgen Tröltzsch, Ikenna Ireka, Dariusz Niedziela, Konrad Steiner, Lothar Kroll

**Affiliations:** 1 Endowed Chair Textile Plastic Composites and Hybrid Compounds, Faculty of Mechanical Engineering, Technical University of Chemnitz, D-09126 Chemnitz, Germany; daisy.nestler@mb.tu-chemnitz.de; 2 Department of Lightweight Structures and Polymer Technology, Faculty of Mechanical Engineering, Technical University of Chemnitz, D-09126 Chemnitz, Germany; juergen.troeltzsch@karlmayer.com (J.T.); lothar.kroll@mb.tu-chemnitz.de (L.K.); 3 Fraunhofer Institute for Industrial Mathematics, D-67663 Kaiserslautern, Germany; iirex4@gmail.com (I.I.); niedziel@itwm.fhg.de (D.N.); konrad.steiner@itwm.fraunhofer.de (K.S.)

**Keywords:** polyurethane foam, rheometry, shear stress, numerical simulation, finite volume method, chemorheology, foam viscosity

## Abstract

Products made of polyurethane foam are manufactured by the chemical reaction of various low-viscosity raw materials and additives. The diversity of different formulations to meet the requirements of the market makes the characterization of their processing and flow properties important for a simple and error-free production. The modeling and simulation of such processes are equally of great importance. This provides additional findings without the expense of real tests and makes it easier to design components. The work described in this paper was carried out against this background. An experimental setup using a rheometer was developed to determine the flow and curing properties of reacting polyurethane foam reproducibly with comparable expansion conditions to industrial processes. The experiment was mathematically modelled to investigate the rheology of reacting polyurethane foams. The mathematical framework consists of coupled, non-linear, partial differential equations for the dynamics and the heat transfer processes in the system. These are solved numerically in 3D using finite volume techniques under adequate physical conditions. The accuracy of two viscosity laws according to the state of the art and their novel combination were investigated in this context. The proposed viscosity model of this study provides accurate results compared to the experiment.

## 1. Introduction

Products made of polyurethane (PUR) foam are present everywhere in daily life. The plastic is available in low-viscosity starting materials for its manufacturing. The forming of PUR products takes place during polymerization. PUR formulations consist of various raw materials and additives. This allows the adjustment of the properties of this plastic according to the requirements. PUR foam is produced through mixing and reacting of these materials. It belongs to the class of reactive plastics therefore and can be flexible to rigid. The main applications of flexible types are automotive seating, crash pads, carpet underlay, bedding, and furniture. Rigid variants are mainly used for building and appliance insulation as well as packaging [1]. The diversity of the chemical composition of PUR formulations for different requirements of the market makes the exact characterization of their processing and flow properties essential for a simple and error-free production. The modeling of this process has a great importance in addition to reliable experimental investigation methods. It provides additional findings without the expense of real tests and makes it easier to design components.

Several experiments for the rheological investigation of reacting PUR foams have been described in the literature. The complexity and difficulty provided certain limitations in the reported studies. This is based on the expansion, curing, temperature development and the fragile foam structure. The undisturbed foam expansion in a test setup, the valid collection of the data and the mathematical evaluation could not be achieved in combination so far. The experimental determination of the viscosity of reacting PUR foam is therefore usually not in the focus. Research has aimed to provide an exact understanding of the creation of the foam structure and its stages. The influence of various process parameters and chemicals were also investigated. Different PUR foam formulations were characterized in order to obtain information on their processing and to compare their rheology.

The plate/plate measuring system was frequently used with the shear rheometer to investigate reacting PUR foams. The great advantage of this experimental setup is that the storage and loss modulus can be determined as essential rheological parameters. It is difficult to ensure undisturbed foam expansion in contrast because of the very small foam volumes that can be investigated. Neff and Macosko used plates with a diameter of 60 mm and a measuring gap of only 2.5 mm [2]. The experiments could be carried out under adiabatic conditions because the plates were temperable. The space between the plates was sealed with a polypropylene ring to keep the PUR foam in between. Storage and loss modulus as well as cell opening were investigated in variation of the stoichiometric ratio of a PUR foam formulation. Bouayad et al. used plates with a diameter of 50 mm at a distance of 1.8 mm [3]. These were not sealed, which caused the foam to continuously flow out. Isothermal test conditions were assumed because of the small plate distance. The test temperature was varied between 25 and 65 °C by heating the plates. Mathematical solutions were presented to describe the flow in the rheometer with radial expansion on the basis of one PUR foam formulation. The characteristic stages of foam creation (induction time, bubble growth, beginning of the polymerization and gelation times) were also investigated using the measurement curves. A strong dependence on the test temperature was proven. Abdessalam et al. used the experimental setup of Bouayad et al. to determine parameters for flow simulation of PUR foam in the production of an automotive underlay carpet [4]. Chandan et al. also investigated the stages of foam creation [5]. He examined different PUR foam formulations. Bakhtiyarov et al. investigated the influence of aluminum particles on the flow properties of PUR foam with a plate/plate measuring system [6]. Kostrzewski used a temperature-controlled capillary rheometer made of aluminum to investigate the flow properties of reacting PUR foam [7]. The reaction was initiated in a lower container with a diameter of 120 mm. Afterwards, the expansion took place up to the capillary with a diameter of 23 mm. The constriction results in a large pressure gradient that was measured and investigated. The test setup is ideally suited for investigating process conditions because of the possibility to determine the temperature, volume flow, and apparent viscosity of the foaming mixture.

There are also various test setups where the PUR foam reacts in a beaker and a measuring body moves constantly in it. This enables investigations with undisturbed foam expansion in the case of large volumes. Webb used a vibrating ball of aluminum, Carriere et al. a pulsating plate of aluminum and Singh used together with Bhattacharya a rotating four-blade vane [8,9,10]. Neff and Macosko noted with regard to this that the geometry of the measuring body and the type of its movement influence the torque measurement values and the exothermic heat of the PUR foam [2]. A coaxial cylinder measuring system with oscillating movement under consideration of the heat absorption of the measuring body is usually preferred for this reason. This was applied by McClusky et al. [11]. They used a glass rod as measuring body, which has a much lower thermal conductivity than metals. The PUR foam reaction took place in a heat-insulated 250 mL beaker. The foam creation process was investigated using one PUR foam formulation. Falke et al. used a torsion rod made of polyvinylchloride (PVC) as measuring body in a similar experimental setup to that of this paper [12]. This was used to analyze and compare different PUR foam formulations with regard to their reaction behavior and foam creation. The setup was used to quantify the flow and curing behavior of a PUR foam formulation under near-application process conditions in this paper. Volume expansion and exothermic temperature development are taken into account and a proposed viscosity model for describing the rheology of the foaming mixture in a simulation was validated with the results.

PUR foam expansion is described from the point of view of model developers by the conversion of a reactive isocyanate-polyol mixture from a low molecular weight resin to a solid foam matrix by heat generation, gas evolution, polymerization, and chain linking. Different approaches exist according to the state of the art to model this. The works of Zhao et al. and Karimi et al. are exemplary mentioned here [13,14]. These include deeply the chemical as well as the physical processes in the manufacturing of PUR foam. The various views on the influence of the gas fraction on the rheology of reacting PUR foam introduces a level of uncertainty in the path to modeling the rheology of foams. McClusky et al. attributed the observed initial drop in viscosity in their experiment to an increase in temperature alone and not to contributions from the gas concentration in the mixture [11]. Neff and Macosko attributed their observed four staged modulus build-up in a reacting PUR foam system to initial bubble expansion, network formation, urea separation and final curing [2]. The commonly adopted material law in the literature for chemorheological fluids proposed by Castro and Macosko specifies the dependence of the mixture rheology on the degree of polymerization and the temperature of the system [15]. Rao et al. assumed that the created gas in the system contributes to the viscosity following the Taylor–Mooney law derived for emulsion [16,17,18]. This model suggests an increase in the mixture viscosity as the gas fraction increases. The proposed mathematical structure of the model in [16] agrees with the descriptions in [2], but it does not account for the initial decrease in the viscosity as in [11] for PUR foams and [19] for polymers. In the same way, the quadratic and quotient models for the dependence of the mixture viscosity on gas fraction presented by Bikard et al. suggest a decrease in viscosity as the gas fraction increased [20]. These models allow the description of the initial decrease in viscosity with contributions from gas fraction and temperature changes [19] but do not consider the increase in viscosity attributed to bubble growth [2].

The approach of the authors of this paper for modeling the PUR foam expansion summarizes the chemical reactions taking place with the Kamal model [21]. No exact information about the chemical composition of PUR foam formulations is required. A basic knowledge of the expansion characteristics is necessary to calibrate model parameters in contrast. This can be determined with free foam experiments in simple geometries. The filling of complex geometries under physically motivated process conditions can be predicted after the calibration of the model. This approach is valuable for foam manufacturers who purchase PUR foam formulations from suppliers that do not provide the exact chemical composition. Computational studies of the authors of this paper about this are presented in [22]. A coupled system of partial differential equations for governing the foam expansion process based on Navier–Stokes equations including equations for heat transfer and degree of polymerization in the system was introduced there. This was solved numerically using finite volume techniques. The simulation results were validated with experiments in tubes. The model described in [22] has also been successfully used to simulate the structural reaction injection moulding (SRIM) with spacer fabrics as inserts [23]. A further extension of the model was carried out in [24] to account for non-uniformity in the expansion of the foaming mixture. The influence of temperature wall conditions on the volume of expanded PUR foam was investigated. The results were compared with experiments in tubes of different diameters and with filling studies in moulds of simple and complex geometry. The temperature-induced non-uniformity in the expansion resulted in a variation of the foam density. This was proven with the computer tomography (CT) of a foam sample, which showed good agreement with the results of macroscopic foam expansion simulations.

The balance between gas creation, heat generation, bubble nucleation and growth as well as phase transition because of the polymerization in PUR foam formation suggests additional contribution of the gas fraction to the mathematical framework of the existing viscosity model [22,24]. Therefore, this study seeks to numerically investigate the influence of the gas volume fraction on the rheology of reacting PUR foams with the aim to validate and improve the existing material models. Results from the simulation are validated with the experiment. The observations indicate a modification of the available rheological models in the literature. This adjustment would account for the contribution of gas fraction at the initial gas creation stage as well as the final gas depletion stage.

## 2. Materials and Methods

### 2.1. Experiment

**Figure 1:**
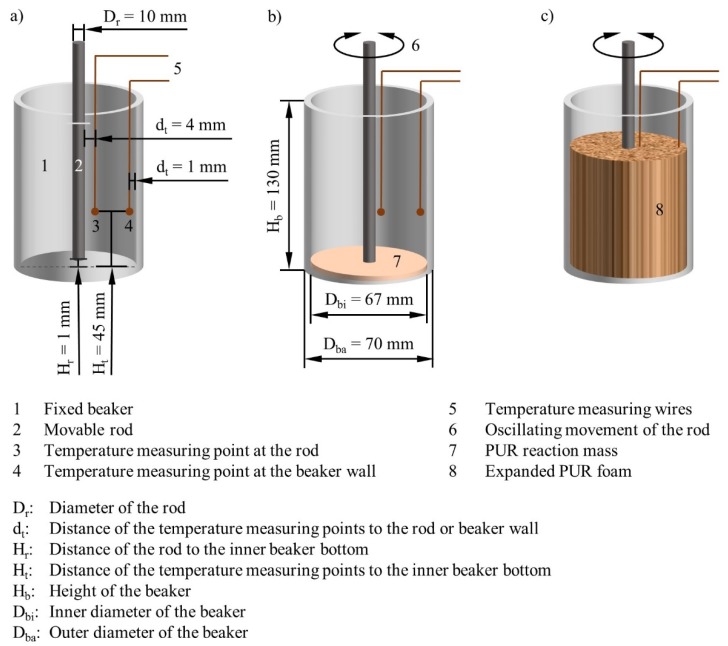
Schematic structure of the coaxial rheometer test for measuring the shear stress and temperature within expanding and curing polyurethane foam (**a**) before and (**b**) after filling in the PUR reaction mass as well as (**c**) after expansion.

A rheometer test setup was designed to characterize the flow properties and curing of expanding PUR foam reaction mixtures (Figure 1). The device Haake Mars III by Thermo Fisher Scientific Inc. (Waltham, MA, USA) was used for this purpose. The Searle principle has been applied. The transfer of the movement from the device to the measuring system was transmitted by polycarbonate (PC) solid rods. An oscillating motion was used to minimize the influence on the unstable foam structure during expansion and curing. The device measures the torque at maximum deflection to maintain the movement when obstructed by the measuring medium. The influences of amplitude and frequency on the measurement results were considered in this study.

Glass beakers with a volume of 400 mL were used as fixed cylinders. The reaction and expansion of the PUR mixture took place in them. The transparency of the glass allows the capturing and analysis of this process. It was recorded with a VP-HMX20C HD camcorder from Samsung Electronics Co., Ltd. (Suwon, Korea). The time of exceeding the volume markings of the beakers was determined in the evaluation of the videos. These had a scale division of 25 mL.

A strong adhesion between the PUR and the cylinders is necessary for the force transmission in the measuring system and the collection of data. This is built up during the polyaddition reaction to both glass and PC. A new beaker and a new PC rod had to be used for each measurement because of this. The measuring system was designed with a large gap between the inner and outer cylinder in order to allow an unimpaired formation of the foam structure. The PC rods had a diameter (*D*_r_) of 10 mm and the beakers an inner diameter (*D*_bi_) of 67 mm. A measuring gap (*H*_r_) of 1 mm was used between the lower circular surface of the PC rod and the inner bottom of the beaker to exclude friction between the cylinders.

The data of the measurements can be used to determine the shear stress within the expanding PUR foam according to the following system of equations. The height of rise of the foam at the PC rod (*h*_f_(*t*)) is calculated with the volume expansion (*V*_f_(*t*)) according to Equation (1):

(1) $$h_{f}\left(t\right)=\frac{4\;V_{f}\left(t\right)}{\pi \;D_{\mathrm{bi}}^{2}}-H_{r}.$$

Using Equation (2), the surface at the PC rod covered with PUR foam (*S*(*t*)) can be calculated:

(2) $$S\left(t\right)=\pi \;D_{r}\;h_{f}\left(t\right)+\;\frac{\pi \;D_{r}^{\;2}}{4}.$$

The force acting on the PC rod (*F*(*t*)) can be determined using the torque measurement data (*M*(*t*)) by Equation (3):

(3) $$F\left(t\right)=\frac{2\;M\left(t\right)}{D_{r}}.$$

The shear stress (*τ*(*t*)) within the expanding PUR foam can be calculated according to Equation (4) finally:

(4) $$\tau \left(t\right)=\frac{F\left(t\right)}{S\left(t\right)}.$$

The tests were carried out at room temperature. To characterize the temperature development during the exothermic PUR reaction, additional measurements were carried out inside the PUR foam. The Testo 735 measuring instrument by Testo SE & Co. KGaA (Lenzkirch, Germany) was used for this purpose. Bending-resistant measuring wires were chosen as temperature sensors. These were installed from above at defined positions in the beaker. Two measuring points were examined. These were each 45 mm above the glass bottom, which corresponds to the 150 mL volume marking of the beakers. One measuring point was at a distance of 4 mm from the PC rod and the other 1 mm from the beaker wall. The temperature measurement curves were recorded at a frequency of 1 Hz.

A reactive 2-component cold foam formulation from Covestro AG (Leverkusen, Germany) was investigated. This produces rigid PUR foam which has free foam weight per volume of about 40 kg/m^3^ according to the data sheet and primarily has closed cells. It consists of the aromatic polyisocyanate Desmodur 44V20 LF (NCO content 30.5–32.5 wt.%) and the polyol formulation BAYDUR 43-101. These contain polyether polyol (OH number 330 mg KOH/g) and all necessary additives for the polyaddition reaction and CO_2_ formation. The two components are processed in a mass ratio of 67 polyol formulation to 100 polyisocyanate. In the rheometer test, the two components were manually mixed and poured into the beaker. The foam formulation has a starting time of 50 s and a tack-free time of 15 min. The time of measurement was determined in accordance with the latter. The time-zero point of all measurement curves is the merging of polyisocyanate and polyol. The influence of the reaction mass on the measurement results was investigated. The maximum mass used was 17 g and corresponds to a free foam volume of 300 mL.

### 2.2. Mathematical Modeling

**Figure 2:**
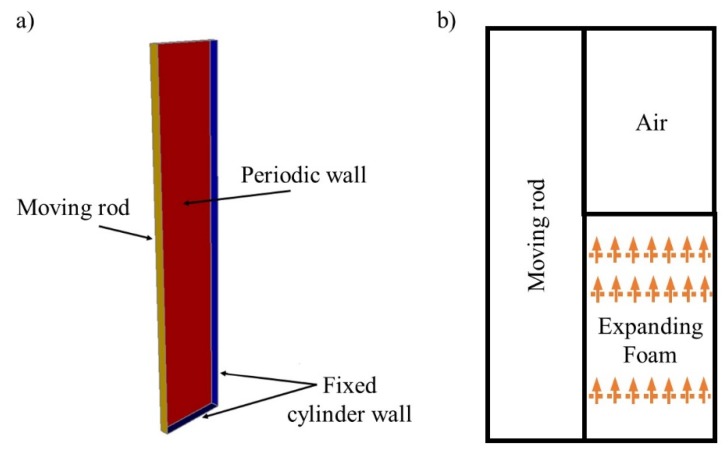
(**a**) 3D slab geometry used in simulations of PUR foam expansion and its torque evaluations and (**b**) setup of the mixed domain used for estimating the thermal condition at the transition area between rod and foam.

The mathematical modeling is based on our own work described and referenced in Section 1. Uniform expansion of the foaming mixture in the domain is assumed. Constant density of the foam is therefore present at any time and spatial position. The system of coupled partial differential equations for foam velocity, pressure, temperature, polymerization, and foam fluid fraction are solved together. Constitutive equations for the foam viscosity, density, thermal conductivity and specific heat together with the appropriate selection of parameters arising in polymerization and temperature equations complete the system. A setup of an inner and outer concentric cylinder with the diameters *D*_r_ and *D*_bi_ was adopted to model and simulate the torque measurement experiments presented in Section 2.1. Both cylinders were assumed to be closed at one end. The reacting pseudo-homogeneous foaming mixture is contained in the gap between them (Figure 1). The outer cylinder is fixed while the inner cylinder oscillates periodically through a pre-defined angle of oscillation and amplitude. The problem domain of Figure 1 is reduced to a rectangular slab cut out from the physical geometry of the problem (Figure 2a). The slab is assumed to be thin enough that curvature effects may be neglected. Periodic boundary conditions are imposed on the state variables on the faces of the slab while no slip velocity conditions were imposed on the walls of the external cylinder. The tangential velocity $v_{\vec{t}}$ at the shear plane caused by the oscillations of the inner cylinder is prescribed by Equation (5), where *A* is the amplitude of oscillation, *ω* is the angular speed, and *t* is time:

(5) $$v_{\vec{t}}=A\;sin\;\left(\omega \cdot t\right).$$

At the walls of the outer cylinder, a heat flow balance is prescribed according to Section 1. The PC rod heats up during foam expansion because of the heat exchange at the transition area with the foam. An additional purely diffusion heat equation problem is solved in order to capture the effect of the temperature change of the rod in the simulation of the rheometer experiment (Figure 1). It is assumed that the heat from the reacting mixture is transferred into the inner cylinder by conduction.

A 2D heat transfer problem in a mixed domain which mimics the physics of the expanding foam is solved to prescribe the temperature condition at the transition area between the inner rod and the reacting foam (Figure 2b). It is assumed that the foam and the air are convected upwards, whereby the local velocity of the expanding foam is estimated from the gradient of the foam height *h*_f_(*t*) from the experiment over time. The heat Equation (6) is therefore solved at every instance in a static mixed domain comprising the foaming mixture, air and the cylindrical rod, where *T* is the temperature, *C_P_* denotes specific heat, *k* thermal conductivity, *ρ* foam density, *H*_R_ heat of reaction parameter, and *ξ* polymerization variable:

(6) $$\rho C_{p}\frac{dT}{dt}=-\nabla \cdot \left(k\nabla T\right)+\rho H_{R}\frac{d\xi }{dt}.$$

The heat source from the chemical reaction (last term in Equation (6)) is accounted for in the region containing the expanding foam alone to solve Equation (7) concerning the degree of polymerization alongside Equation (6) according to Kamal. The following parameters were used for Equation (7): *k*_1_ = 3.0 × 10^−3^ (s^−1^), *k*_2_ = 4.1516 × 10^−3^ (s^−1^) and *m* = 0.5, *n* = 1.5:

(7) $$\frac{d\xi }{dt}=\left(k_{1}+k_{2}\xi^{m}\right){\left(1-\xi \right)}^{n}.$$

**Figure 3:**
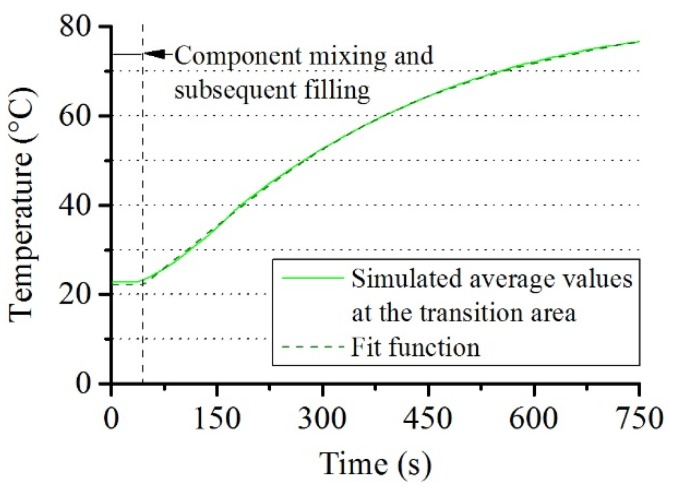
Simulated time averaged temperature values at the transition area between rod and foam as well as an associated fit curve.

In the region consisting of air, Equation (6) has no heat source term because the heat is transferred by conduction and convection in this region. The heat transfer to the rod takes place through the transition areas between rod and foam as well as rod and air resulting in a purely diffuse form of the heat transfer equation in the rod. Appropriate specific heat values corresponding to each material are therefore adopted in each area: in the rod *C*_Prod_ = 1200 (J/kgK), in the air *C*_Pair_ = 1012 (J/kgK), in the foam *C*_Ppur_ = 2000 (J/kgK). For thermal conductivity, *k*_rod_ = 0.20 (W/mK), *k*_air_ = 0.03 (W/mK) and *k*_PUR_ = 0.19 (W/mK) have been used. The continuity of the heat flux is prescribed at each of the transition areas and at the boundaries as stated in Section 1. The average temperature at the transition area between rod and foam is computed at each time (Figure 3).

The fit Equation (8) for the average temperature (*T*_rod_) was constructed in addition, which serves as the boundary condition in the transition area between rod and foam in full 3D foam expansion simulations. These are presented in Section 3.3. *B*_0_ = 40.0950, *B*_1_ = 0.0028, *B*_2_ = −0.2922 and *B*_3_ = 31.9080 are constants obtained from the fitting Equation (8) to the time averaged temperature at the transition area between rod and foam from 2D simulations:

(8) $$T_{rod}=B_{o}tan^{-1}\left(B_{1}\cdot t+B_{2}\right)+B_{3}.$$

## 3. Results and Discussion

### 3.1. Experiment

#### 3.1.1. Amplitude and Frequency Dependence of the Torque

**Figure 4:**
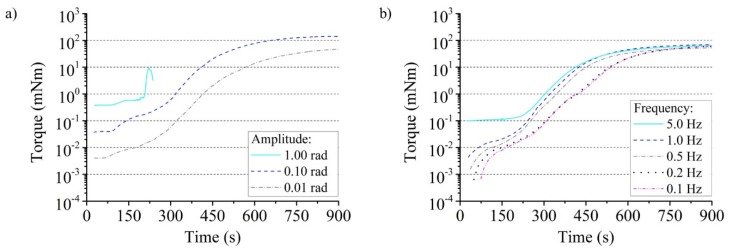
Influence of (**a**) amplitude and (**b**) frequency on torque measurement within reacting PUR foam in the rheometer test setup.

The dependency of the torque from the amplitude was investigated with a deflection frequency of the rod of 1 Hz and a reaction mass of 17 g. If the maximum deflection is increased by a power of ten, the measured torque values also increase by this order of magnitude (Figure 4a). A failure of the foam structure was detected only for an amplitude of 1.00 rad after about 200 s. It ruptured completely from the glass wall in the partially cured state with a cohesive fracture close to the substrate. The excellent adhesion between the PUR and the glass as well as the PC was proven with this. A small amplitude is preferable for characterizing the flow properties and curing of expanding PUR foam reaction mixtures. The formation of micro-pores is less disturbed on the one hand. No load transfer up to the beaker wall can be expected on the other hand, which excludes their influence.

An amplitude of 0.01 rad and a reaction mass of 17 g were used to investigate the relationship between torque and frequency. The dependence could be clearly demonstrated for the experiment, which indicates viscoelastic material behavior of PUR foam during its expansion (Figure 4b). This can be clearly verified at the beginning of the curves. If the foam cures increasingly after about 700 s, it is no longer detectable. The viscous part of PUR foam predominates at low frequencies. Correspondingly low deflection velocities follow the PUR foam without significant hindrance to the measuring rod in its rotation. This results in low torque measurement values. The elastic material behavior is dominant at high frequencies. The inertia of the material and the drive to return to the original shape resist the rotation of the measuring rod and cause higher torque values.

#### 3.1.2. Shear Stress within Reacting PUR Foam and Its Dependence on the Reaction Mass

**Figure 5:**
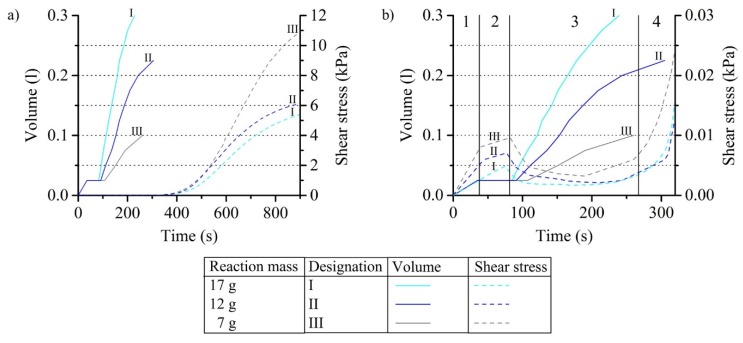
Influence of the PUR reaction mass on the shear stress within reacting PUR foam considering the volume expansion (**a**) during the complete curing time and (**b**) in detail of the first 320 s with four measuring phases: 1—component mixing, subsequent filling in and nucleation, 2—lying time, 3—cell growth and volume expansion, 4—curing.

The influence of the PUR reaction mass on the shear stress was investigated at an amplitude of 0.01 rad and a measuring frequency of 1 Hz. Very low shear stresses below 0.01 kPa arise during foam expansion in the first 250 s (Figure 5a). The curing of the plastic takes place after about 265 s. Shear stresses up to 11 kPa occur subsequently. Detailed analyses of the first 320 s allow the separate identification of individual measurement phases (Figure 5b). The filling of the PUR reaction mass generates a resistance for the movement of the measuring rod, which causes a first increase of the shear stress. This is followed by an almost constant curve which corresponds to the lay time of the reaction mixture. After about 75 s, a drop in shear stress to about 0.005 kPa occurs as the formation of large amounts of the blowing agent CO_2_ takes place. This reduces the mechanical resistance to the movement of the measuring rod and initiates the volume expansion which begins almost simultaneously. Curing of the plastic finally begins after 265 s, which leads to a significant increase of the measurement curves.

The shear stress measurement curves are clearly dependent on the PUR reaction mass, although coverage of the PC rod was taken into account in the stress calculation. The reason for this is the weight per volume of the foam. This varies especially with low PUR reaction masses despite the free expansion in the beaker. 7 g results in 70 kg/m^3^. With 12 and 17 g, a weight per volume of about 55 kg/m^3^ was formed in contrast. More compact foam structures resist the movement of the PC rod stronger and cause higher measuring values. For the characterization of PUR foams in the rheometer test, larger reaction masses are therefore to be preferred in order to measure not in the special range of high free foam weight per volume.

#### 3.1.3. Reproducibility of the Rheometer Test with Reacting PUR Foam

**Figure 6:**
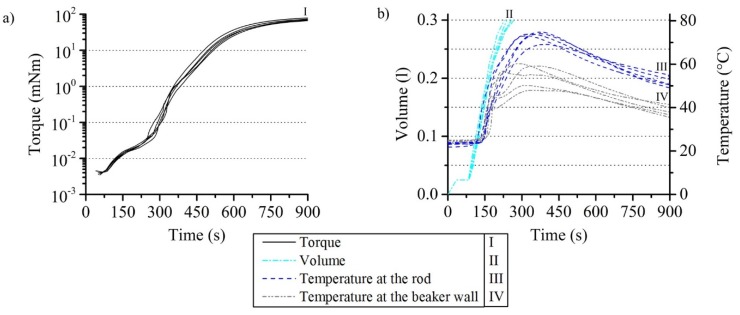
Reproducibility (**a**) of the shear stress within reacting PUR foam and (**b**) the volume expansion as well as the temperature development.

The reproducibility of the results of the rheometer experiment was investigated with an amplitude of 0.01 rad, a frequency of 1 Hz, and a reaction mass of 17 g. The test was repeated five times. The torque measurement curves have an average value of 19.8 µNm with a standard deviation (SD) of 1.5 µNm at their plateau at about 200 s (Figure 6a). The average maximum torque is 71.1 mNm with an SD of 4.8 mNm. Differences between the measurement curves are mainly caused by the manual mixing and filling in of the PUR reaction mass into the beakers. The quality of the mixing and the real mass filled in vary slightly because of this. These facts are also the cause of the slight variations in the volume curves (Figure 6b). The average time to reach the maximum is 250.0 s with an SD of 15.7 s. Deviations in the temperature were primarily caused by a slight displacement of the measuring wires away from their planned measuring points by the expanding PUR foam. The average maximum measured at the rod is 73.0 °C with an SD of 2.3 and 54.9 °C at the beaker wall with an SD of 5.4 °C. A temperature difference at the maximum value of 18.1 °C is therefore present in the test setup. This is comparable to the production of moulded or slabstock foams in industrial processes.

### 3.2. Simulation of the Torque in the Experiment

Plots of the measured torque data suggest a tardy transformation in material property of the foaming mixture (Figure 6a). A slower polymerization of the mixture under the experimental conditions used in this work compared to an adiabatic experimental setup is observed. It is necessary to use lower values for the polymerization parameters in Equation (7) compared to the work described in Section 1 and listed in Section 2.2 for this reason. A slowed polymerization occurs because of a higher heat loss through the outer walls of the beaker used for the torque measurements.

**Figure 7:**
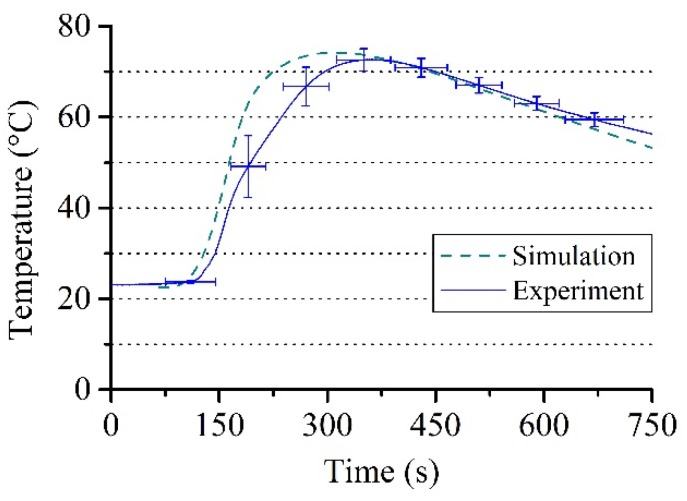
Averaged temperature data of the experiment at the rod with standard deviation compared with the simulated temperature values.

Figure 7 shows a temperature comparison between the measured data at the rod and a simulation carried out. It should be noted that the torque curves in Figure 6 reflect the behavior of the mixture around the rod. The simulated torque values are calculated by multiplying the foam viscosity by tangential velocity gradients obtained from the solution of the system of partial differential equations at rod boundary. The choice of the correct viscosity model is therefore crucial in order to obtain quantitative agreement with experimentally measured values. The approach presented in this study can prove the reliability of the experimental measurement setup used for this paper on the one hand and offers the possibility to validate the foam viscosity models presented in the literature on the other hand.

In this paper, the viscosity model of Equation (9) is used where $\eta_{00}$ is a constant pre-factor and *E_η_* is the activation energy of the polymer blend. ξ represents the degree of polymerization of the mixture and $\xi_{c}$ is the gel point. *H*($\phi_{g}$) describes the contribution of gas fraction to the evolving viscosity of the foaming mixture:

(9) $$\eta_{m}=\eta_{00}exp\left(\frac{E_{\eta }}{RT}\right)\cdot {\left(\frac{\xi_{c}}{\xi_{c}-\xi }\right)}^{C+D\xi }H\left(\phi_{g}\right).$$

It takes the general form of Equation (10):

(10) $$H\left(\phi_{g}\right)=\alpha f\left(\phi_{g}\right)+\beta h\left(\phi_{g}\right).$$

*f*(*ϕ_g_*) is attributed to Bikard et al. and is defined by Equation (11):

(11) $$f\left(\phi_{g}\right)=a_{1}+a_{2}\phi_{g}+a_{3}\phi_{g}^{2}.$$

Equation (12) describes the Taylor–Mooney law for emulsions:

(12) $$h\left(\phi_{g}\right)=exp\left(\frac{H\phi_{g}}{1-\phi_{g}}\right).$$

**Figure 8:**
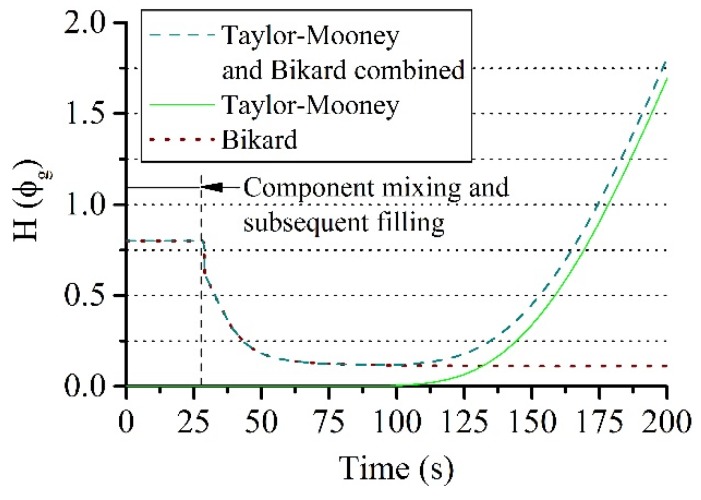
Influence of the gas fraction on the mixture viscosity with the function of Equation (10).

The following parameters were used in the equations listed above: *a*_1_ = 0.8, *a*_2_ = −1.2, *a*_3_ = 0.5, *C* = 1.1, *D* = 1.0, *K* = 1.0. It should be noted that *H*(*ϕ_g_*) = 1 reduces Equation (9) to the Castro–Macosko model. This model specifies the dependence of the material viscosity on the degree of polymerization and the temperature of the system. The dependence on the gas fraction is not taken into account. The viscosity becomes a decreasing function of the gas fraction *ϕ_g_* if *α* = 1 and *β* = 0 in Equation (10). In the case *α* = 0 and *β* = 1, the mixture viscosity increases with rising *ϕ_g_*. A combination of both models from Equations (11) and (12) has also been used in this work with *α* = 1 and *β* > 0. This enables the capturing of the decrease in viscosity when bubble nucleation occurs at the beginning of the process and the increase in viscosity because of bubble growth in the later stages (Figure 8).

### 3.3. Comparison of Experiment and Simulation

**Figure 9:**
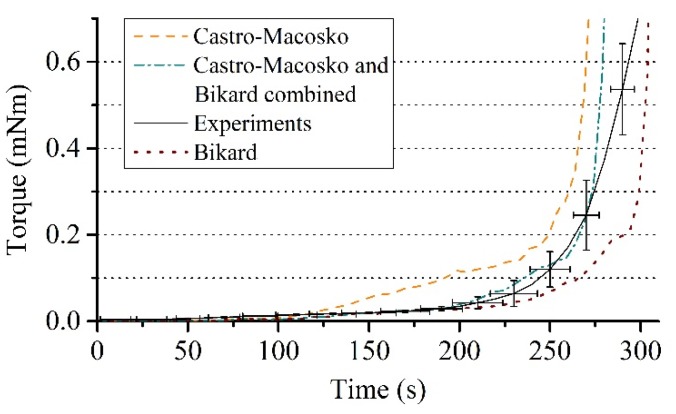
Comparison between the averaged torque measurement curves with standard deviation and the results of the simulation with the Castro-Macosko model, gas fraction model of Bikard et al., and combined gas fraction model with *α* = 1 and *β* = 10^−7^ proposed in this paper.

A reaction mass of 17 g, an amplitude of 0.01 rad and a frequency of 1 Hz were used for the viscosity model (Equation (9)), which are optimized test conditions according to the experiment (Section 3.1). The simulations were performed for up to 250 s where the gelling point is reached. The material expansion is finished at this point, whereby the further measurement process cannot be correctly reproduced in the simulation as the elastic properties of the foam are not captured in this work. Figure 9 shows the comparison of the averaged torque measurement curves of the experiment with the results of the simulations, where the viscosity models of Castro–Macosko, Bikard, and their combination were applied. The best agreement with the experimentally obtained torque curves is shown by the viscosity Equation (9) with gas fraction contribution, which is composed of a combination of Equations (10) and (11). This proves the necessity to account for the contributions from the gas fraction to the viscosity model when considering PUR foams. The mathematical structure of the model of this paper states that the degree of polymerization and variations in temperature alone, described by the Castro–Macosko viscosity model, control the transition of the mixture viscosity. Hence, it leads to a steady increase in the torque values over a time and results in higher values compared to the experimental data.

The influence of the gas fraction model *H*(ϕ_g_) on the torque values was also analyzed in this investigation. Especially the cases corresponding to the model of Bikard (*α* = 1 and *β* = 0), the model of Taylor–Mooney (*α* = 1 and *β* = 10^−7^) and the combination with non-zero (*α* = 1 and *β* = 10^−7^) in Equation (10) were investigated. Thereby, *β* was optimally chosen from several simulations. The respective contributions for each of the cases are shown in Figure 9. The Taylor–Mooney model shows a strong influence of the gas fraction on the mixture viscosity only at the end of the expansion process. The dependency only starts to take effect when the dissolved gas concentration in the liquid has decreased as a result of diffusion into nucleated bubbles or possible evaporation. This agrees with the observed effect of Neff and Macosko [2] on the mixture viscosity that the liquid saturated with gas gradually changes into a mixture with nucleated bubbles. The initial decrease in viscosity as a result of the supersaturation of the gas in the mixture is not captured however. The parameter *β* had to be chosen to generate initial viscosities of its own order (10^−7^ Pas) to obtain correct torque values in later process stages. Initial torque values have been strongly underestimated and are therefore not shown in Figure 9. The Bikard model is able to account for this initial decrease in the mixture viscosity. It has no contribution to the viscosity at the end of the expansion process and does not account for the effect of the depleting dissolved gas on the mixture viscosity compared to the Taylor-Mooney model. As a result, torque values are underestimated at later processing times after 210 s. The combined model was used to take both effects at the beginning and at the end of the expansion process into account. This resulted in the best agreement with the experimentally measured torque values.

**Figure 10:**
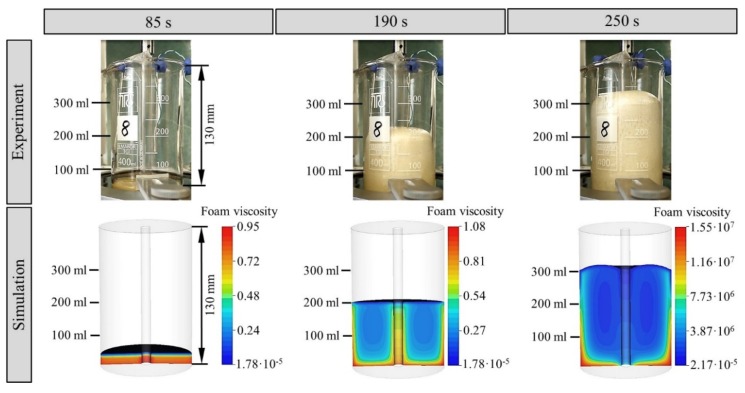
Comparison between real and simulated PUR foam expansion in the rheometer test setup.

The full rheometer test was simulated in 3D with the described mathematical structure in order to further validate the combined viscosity model (Figure 10). The half-elliptical profile of the foam front during expansion shows better agreement with the reality compared to the previous work of the authors of this paper (Section 1) in which the gas contribution of Bikard was used as viscosity model.

## 4. Conclusions

An experimental setup using a rheometer was designed in this work, which is suitable for characterizing the flow and curing behavior of PUR foam formulations. The Searle principle was used as a measuring system. The foam expansion was performed under comparable conditions for the production of molded or slabstock foams in the industry. This refers on the one hand to a large gap for the foam expansion compared to other test setups according to the state of the art. The gap between the cylinders in this study was 28.5 mm. A typical temperature variance for production processes was present within the reacting PUR foam on the other hand. The torque and volume expansion used to calculate the shear stress within the PUR foam was measured in addition to the temperature development. The reproducibility of the experiment was proven. The excellent adhesive strength and the resulting load transfer between the PUR foam and the cylinders were demonstrated by investigating the influence of the amplitude of the torsion rod on the measurement results. Viscoelastic behavior of the PUR foam during its expansion was shown by analyzing the influence of the frequency of the torsion rod. The effect of the PUR reaction mass on the measurement results was also demonstrated. A low foam weight per volume is created when this is very small and gives the torsion rod a higher resistance compared to a higher weight per volume. Optimized test conditions were derived from the findings, which will enable future investigations of different PUR foam formulations and their comparisons. The measurement results were used to validate a PUR foam expansion simulation with the reality.

This paper presents also improvements to a mathematical structure for modeling the PUR foam expansion, which the authors had worked out themselves in previous work. The functional relationship describing the chemorheological properties of the reaction of PUR foam mixtures was investigated for this purpose. Existing models and new configurations were numerically investigated to understand the influence of the degree of polymerization and the contribution of the developed gas to the rheology of expanding PUR foam. The viscosity of reacting PUR foam is characterized by an initial decrease and a final increase over time. This results from the saturation of the liquid with gas at the beginning, which changes into a mixture with nucleated bubbles through polymerization. The combination of the two established rheological models of Bikard and Castro-Macosko was presented in this paper and describes the two phenomena mentioned. The mathematical structure was applied to describe the real test setup using a rheometer in order to validate this. Coupled, nonlinear, partial differential equations describing the dynamics and the heat transfer process in the system were solved numerically using finite volume techniques under adequate physical conditions. The viscosity models of Bikard and Castro-Macosko as well as their combination were comparably applied assuming constant polymerization parameter values and uniform expansion within the system to consider the contribution of the gas fraction. The combination provides the most accurate results compared to the experimental torque measurement data. The improvement of the overall mathematical structure to describe the PUR foam expansion by the new viscosity model was proven by a complete 3D simulation of the experiment using a rheometer. A better agreement of the flow front compared to the earlier work of the authors was found thereby.
